# Reduction of Aflatoxin B_1_ Toxicity by *Lactobacillus plantarum* C88: A Potential Probiotic Strain Isolated from Chinese Traditional Fermented Food “Tofu”

**DOI:** 10.1371/journal.pone.0170109

**Published:** 2017-01-27

**Authors:** Li Huang, Cuicui Duan, Yujuan Zhao, Lei Gao, Chunhua Niu, Jingbo Xu, Shengyu Li

**Affiliations:** 1 School of Environment, Northeast Normal University, Changchun, Jilin, The People's Republic of China; 2 Institute of Agro-food Technology, Jilin Academy of Agricultural Sciences, Changchun, Jilin, The People's Republic of China; Alexandria University, EGYPT

## Abstract

In this study, we investigated the potential of *Lactobacillus plantarum* isolated from Chinese traditional fermented foods to reduce the toxicity of aflatoxin B_1_ (AFB_1_), and its subsequent detoxification mechanism. Among all the investigated *L*. *plantarum* strains, *L*. *plantarum* C88 showed the strongest AFB_1_ binding capacity in vitro, and was orally administered to mice with liver oxidative damage induced by AFB_1_. In the therapy groups, the mice that received *L*. *plantarum* C88, especially heat-killed *L*. *plantarum* C88, after a single dose of AFB_1_ exposure, showed an increase in unabsorbed AFB_1_ in the feces. Moreover, the effects of *L*. *plantarum* C88 on the enzymes and non-enzymes antioxidant abilities in serum and liver, histological alterations of liver were assayed. The results indicated that compared to the control group, *L*. *plantarum* C88 alone administration induced significant increase of antioxidant capacity, but did not induce any significant changes in the histological picture. Compared to the mice that received AFB_1_ only, *L*. *plantarum* C88 treatment could weaken oxidative stress by enhancing the activity of antioxidant enzymes and elevating the expression of Glutathione S-transferase (GST) A3 through Nuclear factor erythroid (derived factor 2) related factor 2 (Nrf2) pathway. Furthermore, cytochrome P450 (CYP 450) 1A2 and CYP 3A4 expression was inhibited by *L*. *plantarum* C88, and urinary aflatoxin B_1_-*N*^*7*^-guanine (AFB-*N*^*7*^-guanine), a AFB_1_ metabolite formed by CYP 1A2 and CYP 3A4, was significantly reduced by the presence of viable *L*. *plantarum* C88. Meanwhile, the significant improvements were showed in histological pictures of the liver tissues in mice orally administered with viable *L*. *plantarum* C88. Collectively, *L*. *plantarum* C88 may alleviate AFB_1_ toxicity by increasing fecal AFB_1_ excretion, reversing deficits in antioxidant defense systems and regulating the metabolism of AFB_1_.

## Introduction

Aflatoxin B_1_ (AFB_1_) is considered to possess the highest toxicity among various types of secondary metabolites produced by a larger number of *Aspergillus* spp, and classified as a Group I carcinogen for humans by the International Agency for Research on Cancer [1]. Many foods such as grains (corn, sorghum, and millet), peanuts, beans, and nuts (almonds, pistachios, etc.) may support the growth of *Aspergillus*, and may be contaminated with aflatoxins. It has been reported that AFB_1_ could induce growth retardation, hepatocellular carcinoma, and immunosuppression [2,3]. Relevant studies indicated that AFB_1_ was predominantly metabolized by cytochrome P450 (CYP 450) enzyme systems after being absorbed in the intestinal tract. Subsequently, under the action of CYP 450, including cytochrome P450 (CYP 450) 1A2 and CYP 3A4, AFB_1_ was transformed to *exo*-AFB_1_-8,9-epoxide (AFBO), which could bind to DNA, proteins, and other critical cellular macromolecules to exert its carcinogenic effect [4]. However, AFB_1_ also could be converted to aflatoxin Q_1_ (AFQ_1_) by CYP 3A4, or aflatoxin M_1_ (AFM_1_) by CYP 1A2, which would be considered one way of detoxification. It was also found that glutathione conjugation could eliminate AFBO through the catalytic action of glutathione S-transferase (GST), which was activated by Nrf2-Antioxidant Response Element (ARE) response [5]. Furthermore, GST A3 appeared to be the critical factor involved in AFB_1_ detoxification in mice [6].

It has been reported that some lactic acid bacteria (LAB) can remove AFB_1_ or have protective effects against AFB_1_. Some relevant studies demonstrated that lactobacilli could inhibit the production of aflatoxin as well as the growth of *Aspergillus* spp [7, 8]. Some researchers also analyzed AFB_1_ removal by lactobacilli *in vitro* and pointed out that lactobacilli could rapidly remove AFB_1_ with a removal rate of approximately 50–80% [9–11]. A study using rat as the animal model suggested that probiotic treatment prevented weight loss and reduced the hepatotoxic effects caused by a high dose of AFB_1_ by increasing the excretion of orally administered aflatoxin *via* the fecal route [1, 12]. Another study reported that use of LAB induced protective effects against the oxidative stress and toxicity of AFB_1_ in part through adhesion [13]. Although lactobacilli can provide protective effects against AFB_1_, the mechanism underlying such protection against AFB_1_ is rarely reported yet, specially the molecular mechanisms of oxidative stress-related pathways.

*L*. *plantarum* C88 (CCTCC NO: M 209254), isolated from Chinese traditional fermented dairy Tofu (a Chinese traditional cheese), has exhibited favorable probiotic properties, including aciduricity, bile resistance and ability to colonize in the gastrointestinal tracts. Our previous experiments showed that *L*. *plantarum* C88 had strong radical scavenging activities, and administration of *L*. *plantarum* C88 significantly improved the antioxidant status of the D-galatose induced oxidatively stress mice [14]. On the basis of these special functions, *L*. *plantarum* C88 seem to have potency against AFB_1_ toxicity. Therefore, in this study we evaluated the detoxification effects of *L*. *plantarum* C88 on AFB_1_ toxicity and examined the underlying mechanisms.

## Materials and Methods

### 2.1 Microorganisms, media, and cultivation conditions

Ten strains of LAB were used in this study, where *L*. *plantarum* C4, C18, C23, C25, C26 and C88 were isolated from Inner Mongolia traditional fermented dairy Tofu and *L*. *plantarum* S2-13, S3-9, S4-7 and S5-16 were isolated and identified from Chinese traditional fermented sauerkraut [14]. All the strains were grown in Man–Rogosa–Sharpe (MRS) broth (Hopebio Co., Qingdao, China) at 37°C for 16 h and stored at 4°C between transfers. The bacteria were harvested by centrifugation (2000 × *g*, 10 min, 4°C), washed twice and resuspended to adjust a density of 10^10^ colony-forming units (CFU)/mL using phosphate-buffered saline (PBS, pH 7.2) (Hopebio Co., Qingdao, China). The viable bacterial samples (10^10^ CFU/mL) were autoclaved at 121°C for 30 min to obtain heat-killed bacterial samples, according to the method described by Choi et al. [15].

### 2.2 AFB_1_ binding assay *in vitro*

The binding assay was performed following the procedure reported by Haskard et al. [10]. The bacterial pellets (viable and heat-killed, 10^10^ CFU/mL) were suspended in AFB_1_ solution (2 μg/mL), and incubated at 37°C for 30 min in a shaking bath (200rpm/min). The bacteria were again pelleted by centrifugation (2000 × *g*, 10 min, 4°C), and the supernatant containing unbound AFB_1_ was collected for high-performance liquid chromatography (HPLC) analysis, which was similar to the analysis described by Samuel et al. [16]. Briefly, the methanol-water (1:1, v/v) was used as the mobile phase at a flow rate of 1 mL/min. Separation was achieved using a C_18_ column (150 mm × 4.6 mm, 5-μm particle size, Agilent, USA) at 30°C with an injection volume of 20 μL. AFB_1_ detection was accomplished using an ultraviolet detector at 360 nm.

### 2.3 *In vivo* assessment of AFB_1_ removal assay

#### 2.3.1 Animals and experimental design

On the basis of *in vitro* AFB_1_ removal assay, *L*. *plantarum* C88 with the highest percentage of binding AFB_1_ was selected for further *in vivo* assessment. Male ICR mice (6 weeks’ old, 18–22 g) were randomly divided into six groups (*n* = 15, per group). All animal procedures were approved by the Institutional Animal Care and Use Committee of Jilin University (SCXK 2015–0001). Animals were housed under standard conditions (25°C, 12 h light/dark cycle, relative humidity 50% ± 5%), and were allowed free access to food and water during the experimental period. After an acclimatization period of 1 week, the viable or heat-killed C88 group respectively received 4.0 × 10^10^ CFU/kg bw (body weight) viable or heat-killed *L*. *plantarum* C88 by gavage, the AFB_1_ group mice received 300 μg AFB_1_/kg bw, in the therapy groups, the same dose of the viable or heat-killed *L*. *plantarum* C88 was administered after 300 μg AFB_1_/kg bw exposure (AFB_1_ + Viable or Heat-killed C88) for 21 days continuously. The control mice received normal saline.

At the end of experimental period (day 21), all the mice were anesthetized by inhalation of diethyl ether, blood samples were collected from retro-orbital venous plexus for the determination. After blood samples were collected, all mice were killed by cervical dislocation. Serum was obtained by centrifugation (2000 × *g*, 10 min, 4°C) and stored at −80°C for further analysis; 10% liver homogenates were prepared by homogenizing frozen tissue in cold normal saline. The homogenates were centrifuged (2000 × *g*, 10 min, 4°C), and the proteins in liver homogenates were determined by the bicinchonininc acid (BCA) protein assay reagent (Dingguo Changsheng Biotech Co., Beijing, China). The remaining part of the liver tissue was divided into two parts: fixed for 48 h in 10% formalin saline for histopathological studies, and stored at −80°C for quantitative real-time polymerase chain reaction (PCR) and Western blot analysis.

#### 2.3.2 Modulation of intestinal absorption, fecal excretion and metabolism of AFB_1_

After the mice were administered AFB_1_ and *L*. *plantarum* C88 on the first day, the feces and urine were collected per hour for 7 h continuously. Then, the AFB_1_ content or the number of lactobacilli in feces was measured. Urine of mice was stored at -20°C, and aflatoxin-*N*^*7*^-guanine levels in urine were measured by HPLC.

AFB_1_ content in fecal samples was measured using the method reported by Mykkänen et al. [17] with slight modifications. Briefly, the feces were frozen using liquid nitrogen, ground into powder, and homogenized with five volumes of benzene-acetonitrile (97:3, v/v). After centrifugation (2000 × *g*, 15 min, 4°C) and filtration, the supernatant containing AFB_1_ was lyophilized, and then the lyophilized powder was dissolved in 500 μL of methanol for HPLC analysis as described at Section 2.2.

To determine the number of lactobacilli in feces, the collected fecal samples were immediately stored at 4°C and analyzed using the method described by Wang et al. [18]. Briefly, the viable lactobacilli in feces were determined by dilution plating with MRS agar medium incubated at 37°C for 48 h. The colonies were identified by classificatory characteristics in morphology, Gram stain and catalase test.

Urine samples were acidified with 1 N HCl and 1 M ammonium formate to pH 5, centrifuged (3000 × *g*, 15 min, 4°C), and then passed through a Sep-Pak C18 cartridges (Waters, USA), and the cartridge was washed with 10 ml of 5% methanol–water. Aflatoxin-*N*^*7*^-guanine were eluted from the cartridge using 40% acidic acetonitrile (acetic acid:acetonitrile: water 1:40:60). The eluant was extracted twice with dichloromethane, and the extracts were lyophilized. Lyophilized powder was dissolved in 30% acetonitrile/ methanol (1:1, v/v) in 20 mM ammonium acetate buffer (pH 3.9) for HPLC analysis. Reversed-phase HPLC with fluorescence detection was used to measure the aflatoxin-*N*^*7*^-guanine level in urine as described by Mykkänen et al. [16].

#### 2.3.3 Antioxidant enzyme assays

Total antioxidant capacity (T-AOC), total superoxide dismutase (T-SOD) activity, glutathione peroxidase (GSH-Px) activity, catalase (CAT) activity, and malondialdehyde (MDA) levels in serum and liver were determined spectrophotometrically using commercially available assay kits (Nanjing Jiancheng Bioengineering Institute, China) following the manufacturer’s protocols.

#### 2.3.4 Histopathological studies

Livers were fixed for 48 h in 10% formalin saline. Tissues were embedded in paraffin and sectioned at 5 μm thickness using a rotary microtome. Sections were stained with hematoxylin-eosin (H&E) for light microscopy examination.

#### 2.3.5 Quantitative real-time PCR analysis

Total RNAs were extracted from tissues using TRIzol reagent (ComWin Biotech Co., Beijing, China) and reverse-transcribed into cDNA using M-MLV reverse-transcriptase (Promega Biotech Co., Beijing, China). Real-time PCR analysis was performed with FastStart Universal SYBR Green Master (Roche Diagnostics GmbH, Mannheim, Germany) and gene-specific forward and reverse primers on a LightCycler 96 Real-Time PCR system (Roche Diagnostics GmbH, Mannheim, Germany). The primers PCR conditions for CYP 1A2, CYP 3A4, GST A3, Nrf2, and β-actin were 95°C for 3 min, 40 cycles of 95°C for 15 s, and 60°C for 1 min. Primer sequences for real-time PCR are shown in Table 1.

**Table 1:** Primer sequences for real-time PCR.

Gene	Primer sequence	NCBI Reference Sequence	References
Nrf2	Forward 5’ - CTTTCAACCCGAAGCACG - 3’ Reverse 5’ - TGGGATTCACGCATAGGA - 3’	NM_010902.3	Present study
GST A3	Forward 5’ - AGATCGACGGGATGAAACTGG - 3’ Reverse 5’ - CAGATCCGCCACTCCTTCT - 3’	NM_001288617.1	Kensler et al. (2014)
CYP 1A2	Forward 5’ - GACGTCAGCATCCTCTTGCT - 3’ Reverse 5’ - GACGTTAGCCACCGATTCCA - 3’	NM_009993.3	Present study
CYP 3A4	Forward 5’ - TCCCTCAACAACCCAGAGGA - 3’ Reverse 5’ - TCAACTCGGTGCTTCTGCTT - 3’	NM_001105159.1	Present study
β-actin	Forward 5’ - TGCTGTCCCTGTATGCCTCTG - 3’ Reverse 5’ - TTGATGTCACGCACGATTTCC - 3’	NM_007393.4	Present study

#### 2.3.6 Western blot analysis

Freshly isolated liver tissue was homogenized in lysis buffer supplemented with protease inhibitors (ComWin Biotech Co., Beijing, China). Nrf2 was extracted using a nuclear fractionation isolation kit (ComWin Biotech Co., Beijing, China). This homogenate was further mixed with buffer [60mM Tris-HCl, 2% sodium dodecyl sulfate (SDS) and 2% β- mercaptoethanol, pH 7.2] and boiled for 10 min. The protein content was determined using BCA protein assay reagent (Dingguo Changsheng Biotech Co., Beijing, China). A 50-μg sample of protein was applied to 10% SDS-polyacrylamide gel electrophoresis and transferred to a nitrocellulose membrane (Millipore, MA, USA) for 90 min. Membranes were blocked with 5% skimmed milk in Tris-buffered saline (TBST) and incubated with primary antibodies, including anti-CYP 1A2, anti-CYP 3A4, anti-GST A3, anti-Nrf2 (Biosynthesis Biotech Co., Beijing, China), and anti-β-actin (ComWin Biotech Co., Beijing, China), for 12 h at 4°C, followed by reaction with horseradish peroxidase-conjugated antibody for 1 h at 37°C. The detected bands were quantified using an image analyzer (ChemiScope 5600, Clinx Science Instruments, Shanghai, China), and β-actin was used as a loading control.

### 2.4 Neutralization

The spleen was aseptically removed from ICR mouse and splenocytes were homogenized in Roswell Park Memorial Institute (RPMI) 1640 medium containing 10% heat-inactivated fetal bovine serum (Hao Yang Biological Manufacture Co., Tianjin, China) and 1% Amphotericin B (MP Biomedicals, France). Erythrocytes were lysed with lysis buffer (Beijing Solarbion Science & Technology Co., Ltd., China) for 1 min at 4°C. Single-cell suspensions (10^6^ cells/well) were cultured in a 6-well culture plate at 37°C, 5% CO_2_ incubator.

To examine the effects of Nrf2 on antioxidant response, neutralization test was performed. 5 mg/ml of purified rabbit anti-mouse Nrf2 monoclonal antibody (Biosynthesis Biotech Co., Beijing, China) or *L*. *plantarum* C88 (10^10^ CFU/ml) were added into splenocytes cells to co-cultivate for 1h. Then, the cells were cultured in the presence of AFB_1_ (2 μg/ml) for 48h. As the positive and negative control (C88 and AFB_1_ groups), splenocytes cells were treated with *L*. *plantarum* C88 or AFB_1_ for 48 h. The untreated splenocytes cells were used as a control. Cell suspensions were centrifuged at 300 × *g* for 5 min at day 2. Supernatants were collected for antioxidant enzyme assays and the cells were used to test the expression of GST A3 by the methods as described at Section 2.3.3–2.3.5. In addition, Western-blot (Section 2.3.5) was used to detect the Nrf2 level in splenocytes at day 2.

### 2.5 Statistical analyses

Experimental data were expressed as means and standard errors (means ± standard error) for each group. Differences between groups were analyzed using one-way analysis of variance (ANOVA), followed by the Tukey post hoc test. *P* value <0.05 was considered to be statistically signifcant.

## Results

### 3.1 The binding ability of bacteria with AFB_1_

**Fig 1:**
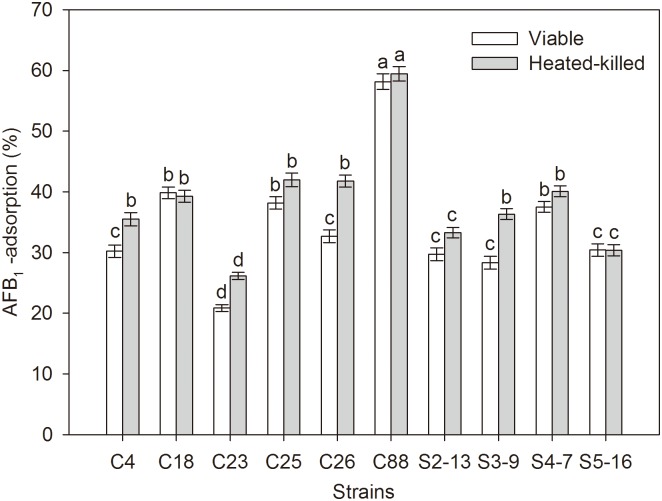
Percentage of AFB_1_ bound to *L*. *plantarum* strains. The results are expressed as mean ± S.D. The different letters in the same rows mean significant difference (p<0.05).

The binding ability of 10 strains with AFB_1_ was examined, and the results are shown in Fig 1. All the strains showed certain binding activities with AFB_1_. The binding rates of different strains (viable and heat-killed bacteria) with AFB_1_ ranged from 20.88% to 59.44%. The differences in binding ability between viable and nonviable cells were not statistically significant in most of the assays, in addition to C4, C26 and S3-9. The heat-killed bacteria showed higher binding ability with AFB_1_ compared with viable bacteria. *L*. *plantarum* C88 demonstrated significantly high binding ability with AFB_1_ compared with the other strains (*P* < 0.05); thus, it was selected for further study.

### 3.2 Reduction of intestinal absorption of AFB_1_

The effects of *L*. *plantarum* C88 on cultivable bacteria and AFB_1_ contents in feces in 7 h after the initial treatments on the first day are shown in Table 2. The number of cultivable bacteria and the concentration of AFB_1_ in the feces were positively associated. Compared with AFB_1_ group, oral administration of viable or heat-killed *L*. *plantarum* C88 significantly increased AFB_1_ contents in feces at each time point (*P* < 0.05). Compared with AFB_1_ group, urinary excretion of AFB-*N*^*7*^-guanine was significantly reduced by the presence of viable *L*. *plantarum* C88. In mice receiving AFB_1_ plus heat-killed *L*. *plantarum* C88, the levels of AFB-*N*^*7*^-guanine were lower than in those receiving only AFB_1_, though those were not statistically significant.

**Table 2:** Effects of *L*. *plantarum* C88 on the cultivable bacteria and AFB_1_ contents in feces and aflatoxin-*N*^*7*^-guanine level in urine.

Group	lactobacilli (log CFU/g feces)				AFB_1_ (μg/g feces)				AFB-*N*^*7*^-Gua (ng/ml urine)
	1h	3h	5h	7h	1h	3h	5h	7h	
Control	8.123±0.152	8.096±0.193	8.060±0.204	8.029±0.196	-	-	-	-	-
AFB_1_	8.125±0.134	8.093±0.215	8.058±0.186	8.033±0.231	1.016±0.063	1.369±0.055	0.828±0.041	0.341±0.038	0.0953±0.004
Viable C88	8.418±0.149	8.258±0.167	8.237±0.229	8.204±0.208	-	-	-	-	-
Heated-killed C88	8.124±0.138	8.094±0.206	8.063±0.233	8.031±0.232	-	-	-	-	-
AFB_1_ + Viable C88	8.421±0.161	8.261±0.185	8.235±0.169	8.206±0.180	2.723±0.051	1.218±0.050	1.183±0.053	1.101±0.039	0.0583±0.005
AFB_1_ + Heated-killed C88	8.123±0.146	8.096±0.218	8.059±0.212	8.032±0.176	3.199±0.056	1.385±0.041	1.345±0.049	1.111±0.045	0.0678±0.008

The results are expressed as mean ± S.D.; each data point is the average of 3 repeated measurements from 3 independently replicated experiments (n = 3).

### 3.3 Alleviation oxidative stress

The effects of different treatments on T-AOC, T-SOD, GSH-Px, CAT, and MDA in serum and liver are presented in Table 3. In the groups only treated with viable or heat-killed *L*. *plantarum* C88, T-AOC, T-SOD, GSH-Px, and CAT activities in serum and liver were increased and MDA content in serum and liver were decreased in comparison with control group. In the therapy groups, mice administered viable or heat-killed *L*. *plantarum* C88 showed the significant increase (P < 0.05) in T-AOC in serum and liver when compared to AFB_1_ treated group. Furthermore, the group orally administrated AFB_1_ plus viable *L*. *plantarum* C88 showed higher T-AOC in serum compared with the control group. The group orally administrated AFB_1_ plus viable *L*. *plantarum* C88 showed the significant increased (P < 0.05) in T-SOD, GSH-Px, and CAT activities in serum and liver compared with the AFB_1_ group. The elevation of MDA content in serum and liver was inhibited after treatment with viable or heat-killed *L*. *plantarum* C88, and this effect seemed to be more pronounced in mice treated with viable *L*. *plantarum* C88.

**Table 3:** Effect of *L*. *plantarum* C88 on the activities of different anti-oxidant enzymes.

Group	Serum					Liver				
	T-AOC (U/mL)	T-SOD (U/mL)	GSH-Px (U/mL)	CAT (U/mL)	MDA (nmol/mL)	T-AOC (U/mg prot)	T-SOD (U/mg prot)	GSH-Px (U/mg prot)	CAT (U/mg prot)	MDA (nmol/mg prot)
Control	12.64±0.57^b^	120.16±2.36^b^	372.13±6.84^b^	1.63±0.21^b^	0.82±0.09^b^	2.65±0.38^a^	3.05±0.30^a^	116.42±2.45^b^	13.40±1.78^b^	0.63±0.05^b^
AFB_1_	7.18±0.46^d^	94.20±2.49^c^	327.23±7.35^c^	0.88±0.16^d^	1.12±0.11^a^	1.12±0.20^c^	1.57±0.26^d^	80.29±3.61^d^	9.24±1.13^c^	0.97±0.08^a^
Viable C88	15.91±0.81^a^	142.34±2.51^a^	398.37±6.44^a^	1.85±0.32^a^	0.54±0.07^c^	2.92±0.26^a^	3.41±0.38^a^	141.66±4.27^a^	20.64±1.64^a^	0.41±0.04^c^
Heated-killed C88	13.13±0.75^b^	124.71±3.17^b^	381.61±6.21^a^	1.67±0.25^b^	0.73±0.09^b^	2.77±0.34^a^	3.23±0.24^a^	132.75±3.14^a^	18.27±2.01^a^	0.55±0.06^c^
AFB_1_ + Viable C88	13.63±0.94^b^	119.31±2.28^b^	356.45±5.18^b^	1.32±0.30^c^	0.92±0.08^c^	1.93±0.25^b^	2.31±0.29^b^	99.51±3.21^c^	11.51±1.62^b^	0.76±0.05^b^
AFB_1_ + Heated-killed C88	10.22±0.69^c^	96.83±1.33^c^	331.03±5.55^c^	0.98±0.17^d^	1.07±0.09^b^	1.70±0.31^b^	1.92±0.28^c^	84.04±2.95^c^	10.88±1.50^c^	0.85±0.04^a^

The results are expressed as mean ± S.D.; each data point is the average of 3 repeated measurements from 10 independently replicated experiments (n = 10).

The different letters in the same rows mean significant different (p<0.05).

### 3.4 Histopathological studies

**Fig 2:**
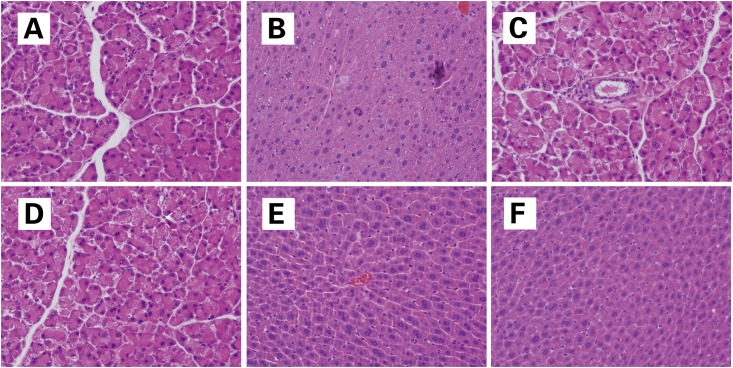
Photomicrographs of hepatic tissue of mice. (a) Hepatic tissue of mice in the control group, with the normal histological structure of liver lobule; (b) hepatic tissue of mice in the AFB_1_ group, with loss of intact liver plates, cytoplasmic vacuolization, cytolysis, and necrosis of hepatocytes; (c) mice treated with viable C88 showing normal hepatocytes and portal tract; (d) mice treated with heat-killed C88 showing nearly normal hepatocytes and portal tract; (e) hepatic tissue of mice in the AFB_1_ plus viable *L*. *plantarum* C88 therapy group, with alleviation of cytoplasmic vacuolization and no necrosis of hepatocytes; (f) hepatic tissue of mice in the AFB_1_ plus heat-killed *L*. *plantarum* C88 therapy group, with less hepatic injury. H&E staining was used (magnification 400×).

No serious tissue damage in the liver was observed in viable and heat-killed *L*. *plantarum* C88 groups. AFB_1_ exposure caused significant damage to the liver, including loss of hepaticcord, cytoplasmic vacuolization, and necrosis of hepatocytes. AFB_1_ and viable *L*. *plantarum* C88 co-treatment group significantly alleviated such hepatic injury, whereas the histological changes in the AFB_1_ plus heat-killed *L*. *plantarum* C88 group was not so evident (Fig 2). These results indicate that C88 supplementation mitigates AFB_1_ induced liver injury.

### 3.5 Inhibition of CYP 1A2 and CYP 3A4 expression

**Fig 3:**
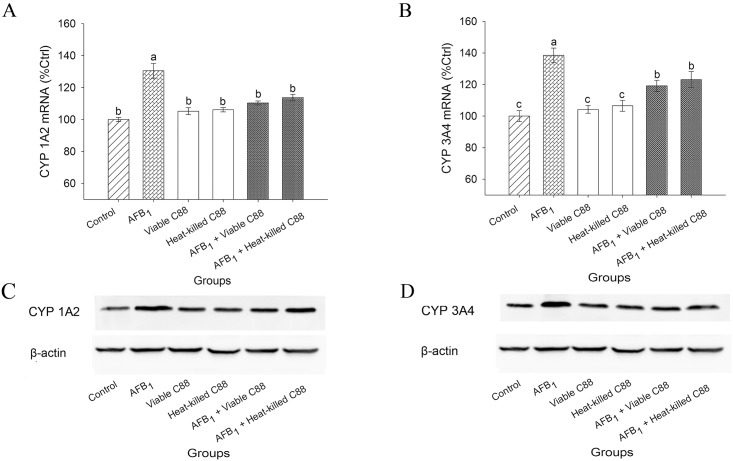
Effect of *L*. *plantarum* C88 on reduction of biotransformation of AFB_1_ *via* inhibition of CYP 1A2 and CYP 3A4 expression. CYP 1A2 (A) and CYP 3A4 (B) gene expression was measured by RT-PCR. Expression of CYP 1A2 (C), CYP 3A4 (D) and β-actin was detected by Western blot analysis. β-actin was used as a housekeeping control. The results are expressed as mean ± S.D.; each data point is the average of 3 repeated measurements from 10 independently replicated experiments (n = 10). The different letters in the same rows mean significant difference (p<0.05).

To confirm whether the biotransformation of AFB_1_ could be inhibited by *L*. *plantarum* C88, the expression of CYP 1A2 and CYP 3A4 was detected (Fig 3). After co-treatment with AFB_1_ and viable *L*. *plantarum* C88, a significantly decreased expression of CYP 1A2 and CYP 3A4 could be observed compared with the AFB_1_ group. Down-regulation of CYP 1A2 and CYP 3A4 was presumed to be a protective mechanism different from the activation of detoxification. The results of Western blot confirmed the aforementioned findings.

### 3.6 Upregulation of GST A3 and Nrf2 expression

**Fig 4:**
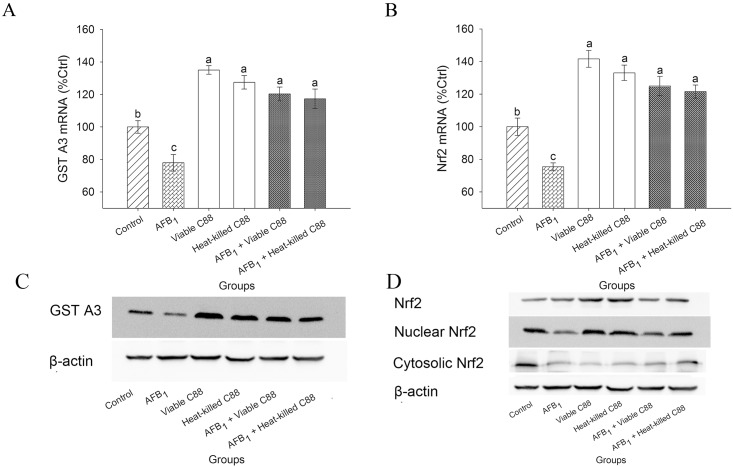
Effect of *L*. *plantarum* C88 on detoxification of AFB_1_ *via* upregulation of GST A3 and Nrf2 expression. GST A3 (A) and Nrf2 (B) gene expression was measured by RT-PCR. Expression of GST A3 (C), Nrf2, Cytoplasmic Nrf2 and Nuclear Nrf2 (D) and β-actin was detected by Western blot analysis. β-actin was used as a housekeeping control. The results are expressed as mean ± S.D.; each data point is the average of 3 repeated measurements from 10 independently replicated experiments (n = 10). The different letters in the same rows mean significant difference (p<0.05).

The expression of GST A3 that could catalyze the conjugation of glutathione (GSH) and AFBO was also evaluated. *L*. *plantarum* C88 significantly increased the expression of GST A3, with maximum expression in the group that received only viable *L*. *plantarum* C88 (Fig 4). The combined treatment of AFB_1_ and *L*. *plantarum* C88 succeeded to induce a significant increase in expression of GST A3 toward the AFB_1_ group. To investigate whether the increase in the expression of GST A3 was linked with the upregulation of Nrf2 expression, the expression of Nrf2 genes was further evaluated in the liver of mice. The results indicated that Nrf2 expression was highly increased in AFB_1_ + *L*. *plantarum* C88 treatment groups compared with that in the AFB_1_ group; treatment with only viable or heat-killed *L*. *plantarum* C88 increased Nrf2 level significantly (P < 0.05), and this increase was pronounced in the group received the viable *L*. *plantarum* C88; Western blot analysis confirmed the results. As the activation of Nrf2 depends on its nuclear translocation, the expression of Nrf2 was assessed in nuclear and cytoplasm fractions. After oral administration of viable and heat-killed *L*. *plantarum* C88, the expression of Nrf2 in nuclear fraction was significantly increased, indicating a nuclear translocation from cytoplasm.

### 3.7 Reducing oxidative stress through modulating the Nrf2 signaling pathway

**Fig 5:**
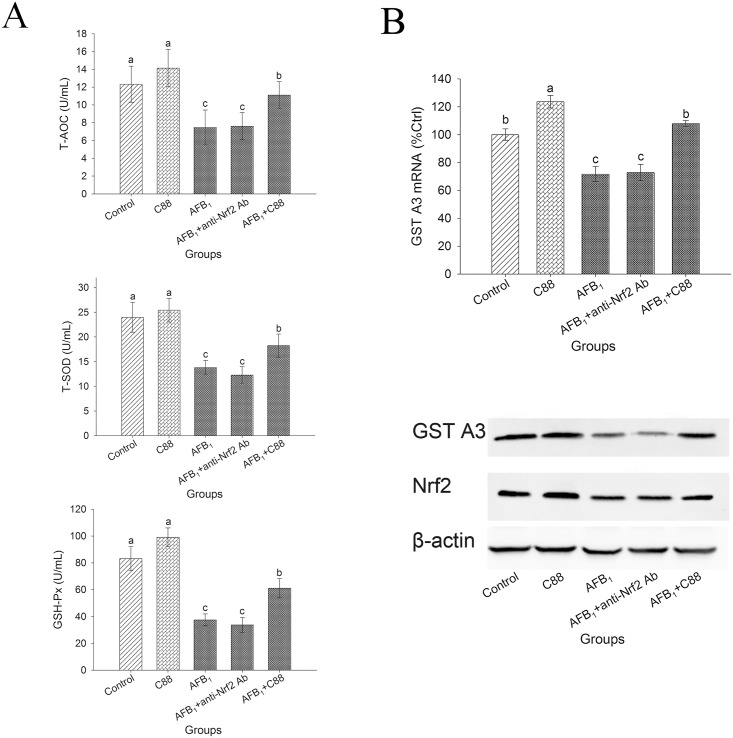
Reducing oxidative stress through modulating the Nrf2 signaling pathway. Anti-mouse Nrf2 monoclonal antibody or *L*. *plantarum* C88 (10^10^ CFU/ml) were added at the beginning of the culture period. Splenocytes were cultured in the absence (control or C88) or presence (AFB_1_) of 2 μg/ml AFB_1_. T-AOC, T-SOD, GSH-Px activities in cell culture supernatants (A). GST A3 gene expression in splenocytes was measured by RT-PCR. Expression of GST A3, Nrf2 and β-actin was detected by Western blot analysis. β-actin was used as a housekeeping control (B). The results are expressed as mean ± S.D.; each data point is the average of 3 repeated measurements from 10 independently replicated experiments (n = 10). The different letters in the same rows mean significant difference (p<0.05).

The aim of neutralization test was to clarify whether upregulation of GST A3 and enhancement of antioxidant enzyme activity were specific associated with the Nrf2 signaling pathway. As compared to the control group, administration of AFB_1_ not only markedly decreased the activities of different antioxidant enzymes, but also down-regulated the expression of GST A3 and Nrf2 (P < 0.05), but the reverse trend was found in the group only received *L*. *plantarum* C88 supplementation. Pretreatment with *L*. *plantarum* C88 enhanced the antioxidant capacity in supernatants and elevated the expression of GST A3 mRNA in splenocytes. With addition of anti-Nrf2 antibody, Nrf2 level was significantly decreased (P < 0.05), and the activities of different antioxidant enzymes were depressed (Fig 5A), indicating that Nrf2 strongly involved in increasing antioxidant capacity. Furthermore, the addition of anti-Nrf2 antibody significantly (P<0.05) decreased expression of GST A3 (Fig 5B).

## Discussion

Previous studies have shown that lactobacilli have the ability to efficiently remove AFB_1_, but the specific detoxification mechanism is poorly understood [9–11]. This study may be the first to explore the protective mechanisms of the oral administration of lactobacilli against AFB_1_ chronic toxicity in mice. In this study, *L*. *plantarum* C88 was selected, which presented the highest binding ability with AFB_1_ using AFB_1_ binding assay *in vitro* compared with other strains. Furthermore, both viable and heat-killed *L*. *plantarum* C88 showed strong AFB_1_ binding activity, which guaranteed that *L*. *plantarum* C88 could exert better effects regardless of lower pH or high levels of bile salts. The presence of bile salts and intestinal tract pH affected the AFB_1_ binding ability of viable bacteria [19]. This is not inexplicable; a previous study pointed out that the binding activities of heat-killed bacteria were not drastically changed compared with those of viable bacteria because heat treatment might change the original binding site of the viable bacteria but expose new binding sites [20].

To further explore the detoxification effects of *L*. *plantarum* C88 on AFB_1_, an *in vivo* experiment using a mouse model was conducted with initial treatments on the first day. The presence of unabsorbed AFB_1_ and the number of lactobacilli in the feces showed a positive correlation, suggesting that *L*. *plantarum* C88 was able to retain additional AFB_1_ inside the intestinal lumen most probably by binding AFB_1_ to the bacterial surface. Moreover, the highest levels of fecal AFB_1_ and number of lactobacilli were observed at the second hour post dose, suggesting that probiotic aflatoxin binding occurs immediately after administration of AFB_1_. The present findings indicated that *L*. *plantarum* C88 might act as a biological barrier in the intestine under normal conditions, thereby reducing the bioavailability of AFB_1_ ingested orally and hence avoiding its toxic effects. Previous studies have demonstrated that *L*. *rhamnosus* GG could modulate intestinal AFB_1_ absorption in rats by increasing fecal AFB_1_ excretion [21]. Oral administration of 2 × 10^10^ CFU per day of *L*. *rhamnosus* LC705 in humans was followed by an increase in the fecal counts of lactobacilli [22].

The histological results reported in the current study confirmed the biochemical results and indicated that AFB_1_ induced severe histological changes in the liver of mice. Similar histopathological changes of AFB_1_ induced hepatic damage have been previously reported [23,24]. The significant recovery of hepatic tissues in mice treated with AFB_1_ combined with viable *L*. *plantarum* C88, as demonstrated in the histopathological sections (Fig 2D), was consistent with previous results in mice [24]. The result obviously demonstrate the potential beneficial effects of *L*. *plantarum* C88 to counteract the oxidative stress induced by AFB_1_.

Oxidative stress is a critical mechanism contributing to initiation and progression of hepatic damage caused by AFB_1_ toxicity by increasing lipid peroxidation and decreasing activities of antioxidant enzymes. T-AOC reflects the capacity of the nonenzymatic antioxidant defense system. SOD, GSH-Px, and CAT are thought to play a crucial role in the interception and degradation of superoxide anion and hydrogen peroxide. MDA is an end product and indicator of the lipid peroxidation process, which can react with biomolecules and exert cytotoxic and genotoxic effects. Viable and heat-killed *L*. *plantarum* C88 supplementation in the diet improved the activities of antioxidant enzymes and decreased lipid peroxidation in serum and liver. After feeding of viable and heat-killed *L*. *plantarum* C88 along with AFB_1_, the significantly increased T-AOC, SOD, GSH-Px, and CAT activities, and decreased lipid peroxidation implied that *L*. *plantarum* C88 might reverse the oxidative damage caused by AFB_1_ (Table 2). An *in vivo* study showed that probiotic fermented milk containing *L*. *rhamnosus* GG and *L*. *casei* Shirota ameliorated hepatic damage induced by AFB_1_ in rats by enhancing the activities of antioxidant enzymes [25]. *L*. *casei* and *L*. *reuteri* significant decreased lipid peroxidation in liver and kidney against oxidative stress in rats fed aflatoxin-contaminated diet [24]. Furthermore, previous studies indicated the elevation of antioxidant enzymes was triggered by the activation of Nrf2 signaling pathway [26]. In agreement with previous findings, we found a strong positive correlation between the activity of antioxidant enzymes and the level of Nrf2 in splenocytes cells, and suggest that Nrf2 may be capable of interacting positively to contribute to reduced oxidative stress triggered by AFB_1_.

It has been reported that AFB_1_ could be converted to AFQ_1_ and AFBO by CYP 3A4, or AFM_1_ and AFBO by CYP 1A2, to finally exert its carcinogenic effects. CYP 3A4 expression level was the most important determinant of the AFB_1_ disposition toward these primary metabolites [27]. AFBO reacts with nucleophilic centers in the DNA and proteins, forming covalently bound aflatoxin-*N*^*7*^-guanine (AFB_1_-*N*^*7*^-Gua) and lysine adducts. AFB_1_-DNA adduction is believed to be the source of point mutations that initiate AFB_1_-induced hepatocarcinogenesis [28]. The present study found that the CYP 1A2 and CYP 3A4 expression levels were decreased on administration of *L*. *plantarum* C88. Furthermore, we evaluated the urinary excretion of AFB-*N*^*7*^-guanine, and urinary AFB-*N*^*7*^-guanine was significantly reduced by presence of *L*. *plantarum* C88 (Table 2). This finding indicated that *L*. *plantarum* C88 could down-regulate the expression of CYP 1A2 and CYP 3A4 at the transcription level which further reduced AFBO concentration. Very few studies have been performed so far on the protective effects of lactobacilli against AFB_1_ by influencing the CYP 450 pathway. Only Gratz et al. [20] reported that *L*. *rhamnosus* GG reduced AFB_1_ availability *in vitro* by down-regulating the expression of CYP 3A4. Therefore, the present findings might provide new thought for the reduction of AFB_1_ toxicity by LAB.

A relevant study confirmed that AFBO could be catalyzed by GST to conjugate with GSH to form a water-soluble AFB_1_-GSH, which was subsequently excreted in the bile and urine [23]. As GST A3, a member of GST family, appeared to be the critical factor involved in AFB_1_ detoxification in mice, the expression of GST A3 was measured in mice fed *L*. *plantarum* C88; the significantly increased GST A3 level implied that *L*. *plantarum* C88 might afford significant protection against AFBO, which could further induce DNA damage and protein denaturation. *L*. *fermentum* I5007 has been shown to increase the level of detoxifying GST in Caco-2 cells [29]. Moreover, the mechanism of GST A3 activation is required to be explored. Nrf2 signaling pathway is involved in triggering the expression of many genes encoding for detoxification, cytoprotective and antioxidant enzymes. In a previous study, following oral treatment with AFB_1_, the downregulation of GST A3 and an increase of AFB_1_–DNA adducts level were observed in Nrf2 knockout mice [30]. In the present study, as shown in Real-time PCR result, after the addition of anti-Nrf2 antibody, GST A3 mRNA level was declined due to the formation of antigen antibody complex, indicating that the changes of GST A3 level depends on Nrf2 level. Therefore, we speculated that GST A3 was adjusted through Nrf2 pathway, and Western-blot result verified this speculation.

## Conclusions

**Fig 6:**
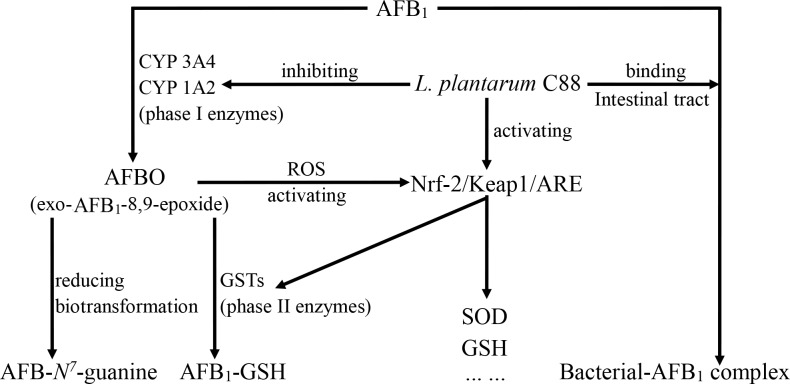
Proposed pathways of protection by *L*. *plantarum* C88 against AFB_1_ toxicity. Protection mechanisms of *L*. *plantarum* C88 against AFB_1_ toxicity *in vivo* are associated with increasing fecal AFB_1_ excretion, decreasing AFB_1_ epoxidation catalyzed by CYP 1A2 and CYP 3A4, coupled with enhancing the activities of different antioxidant enzymes and GST detoxification which are connected with the Nrf2 signaling pathways.

*L*. *plantarum* C88, a selected lactobacillus with good AFB_1_-binding ability *in vitro*, can increase fecal AFB_1_ excretion, reduce lipid peroxidation, and reverse deficits in antioxidant defense systems to alleviate AFB_1_ toxicity. *L*. *plantarum* C88 might play a role in the suppression of CYP 1A2 and CYP 3A4 expression to decrease the production of AFBO and activate GST A3 through Nrf2 signaling pathways to improve glutathione-conjugating activity and hence induce detoxification (Fig 6).

## Supporting Information

S1 Fig Effect of *L*. *plantarum* C88 on aspartate aminotransferase (ALT) and alanine aminotransferase (AST). The results are expressed as mean ± S.D (n = 10). The different letters in the same rows mean significant difference (p<0.05).
(TIF)

S1 Table Effect of *L*. *plantarum* C88 on body weight gain and feed intake. The results are expressed as mean ± S.D (n = 15). The different letters in the same rows mean significant difference (p<0.05).
(DOCX)
