# Supplementary figures and images for: Reduction of Aflatoxin B1 Toxicity by Lactobacillus plantarum C88: A Potential Probiotic Strain Isolated from Chinese Traditional Fermented Food “Tofu”
Source: PLoS One. 2017 Jan 27;12(1):e0170109. doi: 10.1371/journal.pone.0170109 (PMC5271326; doi:10.1371/journal.pone.0170109)

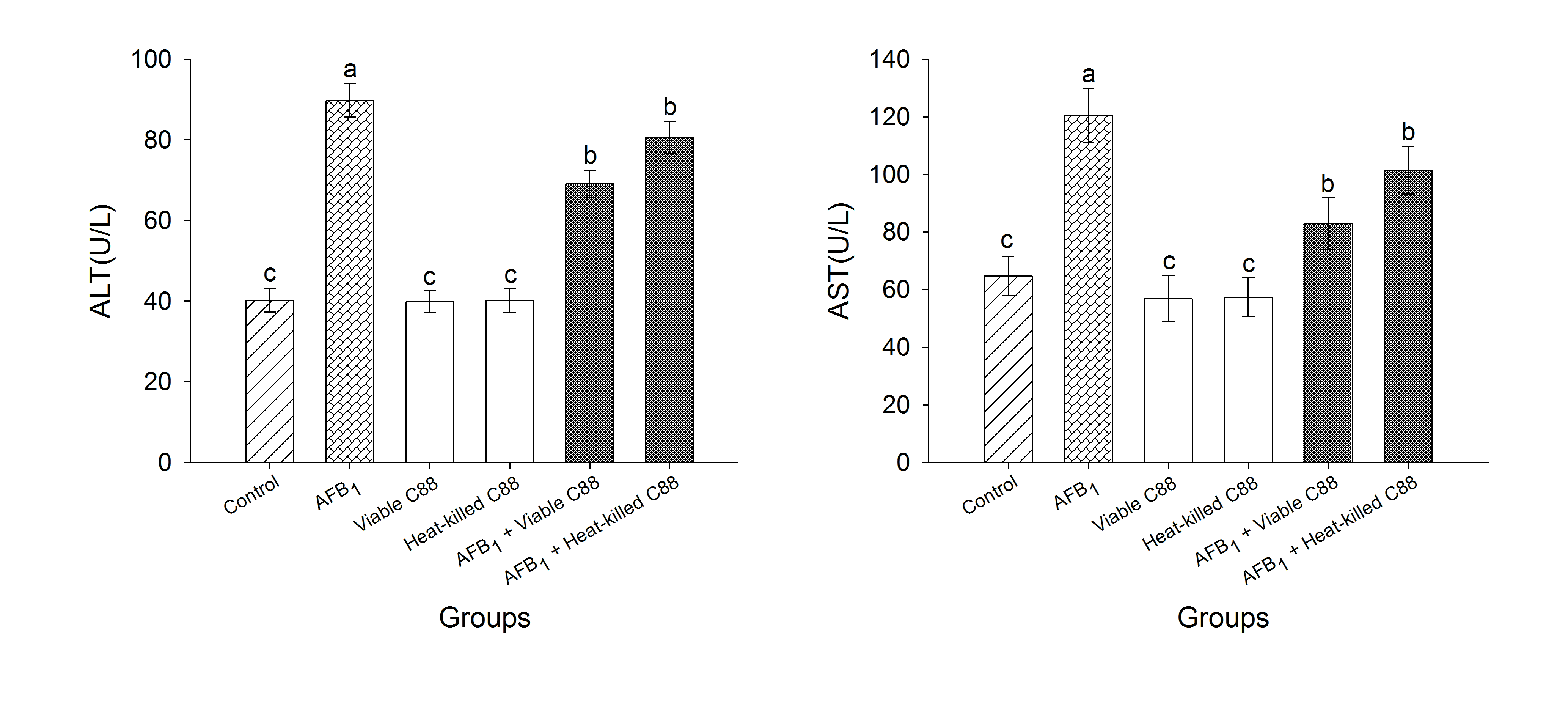

Supplement: S1 Fig — The results are expressed as mean ± S.D (n = 10). The different letters in the same rows mean significant difference (p<0.05). (TIF) [file pone.0170109.s001.tif]
