# Supplementary material for: Reduction of Aflatoxin B1 Toxicity by Lactobacillus plantarum C88: A Potential Probiotic Strain Isolated from Chinese Traditional Fermented Food “Tofu”
Source: PLoS One. 2017 Jan 27;12(1):e0170109. doi: 10.1371/journal.pone.0170109 (PMC5271326; doi:10.1371/journal.pone.0170109)
Supplement: S1 Table — The results are expressed as mean ± S.D (n = 15). The different letters in the same rows mean significant difference (p<0.05). (DOCX) [file pone.0170109.s002.docx]

S1 Table. Effect of *L. plantarum* C88 on body weight gain and feed intake.

| Group | Body weight (g) | | | Total feed intake (g) | Total weight gain (g) |
| --- | --- | --- | --- | --- | --- |
|  | 1 day | 10 day | 21 day |  |  |
| Control | 20.03±0.74^a^ | 28.31±1.95^a^ | 34.76±4.79^b^ | 2198 | 14.73±2.05^b^ |
| AFB_1_ | 20.05±0.83^a^ | 27.62±2.13^b^ | 30.48±4.61^c^ | 1829 | 10.43±1.85^c^ |
| Viable C88 | 19.89±0.95^a^ | 29.78±2.67^a^ | 36.82±3.88^a^ | 2489 | 16.93±2.04^a^ |
| Heated-killed C88 | 20.14±0.66^a^ | 29.92±2.15^a^ | 37.09±4.20^a^ | 2456 | 16.95±2.33^a^ |
| AFB_1_ + Viable C88 | 19.96±0.99^a^ | 28.29±2.34^a^ | 32.99±2.95^b^ | 2035 | 13.03±2.82^b^ |
| AFB_1_ + Heated-killed C88 | 20.08±0.72^a^ | 28.11±2.94^a^ | 31.17±2.53^c^ | 1814 | 11.07±1.74^c^ |
